# Stimulus salience conflicts and colludes with endogenous goals during urgent choices

**DOI:** 10.1016/j.isci.2023.106253

**Published:** 2023-02-21

**Authors:** Emily E. Oor, Terrence R. Stanford, Emilio Salinas

**Affiliations:** 1 Department of Neurobiology & Anatomy, Wake Forest School of Medicine, Winston-Salem, NC 27157, USA

**Keywords:** Behavioral neuroscience, Cognitive neuroscience

## Abstract

Selecting where to look next depends on both the salience of objects and current goals (what we are looking for), but discerning their relative contributions over the time frame of typical visuomotor decisions (200–250 ms) has been difficult. Here we investigate this problem using an urgent choice task with which the two contributions can be dissociated and tracked moment by moment. Behavioral data from three monkeys corresponded with model-based predictions: when salience favored the target, perceptual performance evolved rapidly and steadily toward an asymptotic level; when salience favored the distracter, many rapid errors were produced and the rise in performance took more time—effects analogous to oculomotor and attentional capture. The results show that salience has a brief (∼50 ms) but inexorable impact that leads to exogenous, involuntary capture, and this can either help or hinder performance, depending on the alignment between salience and ongoing internal goals.

## Highlights

• Stimulus salience and internal goals negotiate the next target to look at for ∼50 ms
• The involuntary capture of attention by stimulus salience is brief but inexorable
• The search for a visual target can be helped or hindered by stimulus salience
• Looking away from a salient target is akin to looking toward an inconspicuous one

## Graphical abstract

**Figure:**
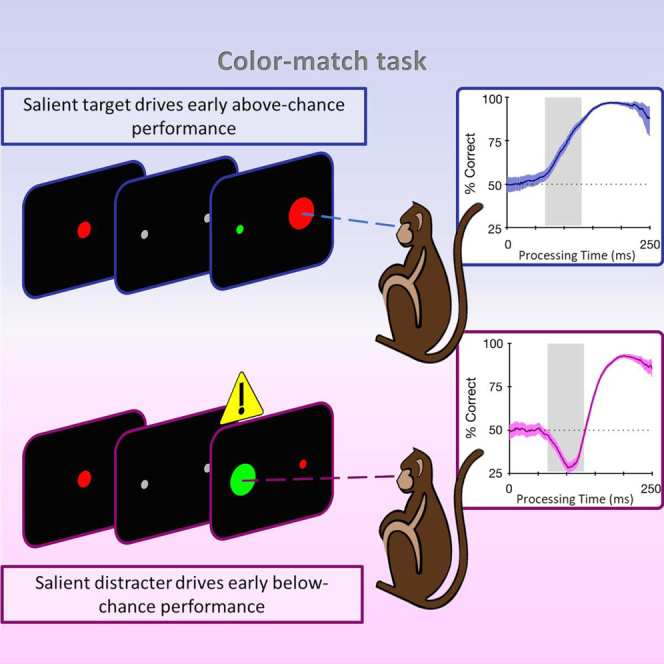

## Introduction

Saccadic choices result from dynamic interplay between exogenous (or bottom-up) and endogenous (or top-down) signals derived, respectively, from the physical properties of visual objects and one’s internal goals.^1^^,^^2^^,^^3^ Although the qualitative distinctions between these mechanisms are well established, pinpointing their specific, real-time contributions to normal visual scanning has been more challenging. For instance, salience definitely influences visual search behavior when it is explicitly guided by bottom-up information,^4^^,^^5^ but during goal-directed, everyday-life tasks (e.g., making a sandwich), its contribution is much more muted or may seem to vanish altogether.^6^^,^^7^^,^^8^ Furthermore, studies aiming to precisely outline the conditions that lead to attentional or oculomotor capture, wherein task-irrelevant stimuli involuntarily draw attention or gaze itself, have revealed many subtle complexities.^9^^,^^10^

Importantly, in many of the prior studies on the topic, performance was associated with relatively long reaction times (**RTs**), much longer than the 200–250 ms intersaccadic intervals that characterize normal visual scanning.^11^ The interaction between exogenous and endogenous signals is likely to have manifested in relatively subtle, indirect ways in those studies because there are indications that this interaction evolves at a much more rapid timescale on the order of a few tens of milliseconds.^12^^,^^13^ The evidence comes from so-called “urgent” tasks, with which the development of a subject’s choice can be tracked moment-by-moment.^14^^,^^15^ Using this approach, we previously found that even when the goal is to look away from a cue stimulus (antisaccade task), its onset triggers a brief but potent exogenous response that promotes an erroneous, involuntary saccade to the cue.^12^^,^^16^ These and other data^13^ suggest that to rigorously characterize the impact of salience on saccadic choices, it is critical to determine the time course of its influence relative to that of the endogenous signals that steer the eyes toward predefined goals.

To do this, we designed an urgent choice paradigm in which the relative salience between a target and a distracter varies (via size differences) but is not tied to the goal of the task (defined via a color-match rule). The behavior in this task was consistent with model-based expectations: when the exogenous signal favored the target, choice accuracy rose early and saturated quickly; in contrast, when the same signal favored the distracter, many rapid errors were produced and it took more time for accuracy to rise above chance—just as in the antisaccade task^12^^,^^16^ but without any instruction to look away. The results show (1) that exogenous events have brief, stereotypical effects that can either help or hinder visuomotor performance and (2) that assessment of their relevance to ongoing goals occurs extremely rapidly as a normal part of the saccadic choice process.

## Results

### Perceptual performance in two urgent tasks suggests a temporally constrained role for salience during saccadic choices

In previous studies, we measured the time-dependent contribution of perception to saccadic choices in two tasks, an urgent color-match task and an urgent antisaccade task. Key differences and similarities between them suggested a specific hypothesis about the functional contribution of salience to oculomotor behaviors. We summarize the essential findings and how they led us to the current experiment.

**Figure 1:**
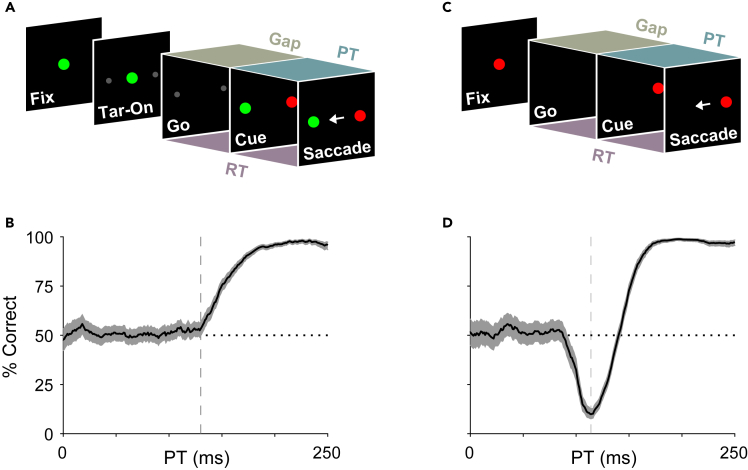
Perceptual performance in two urgent tasks suggests a temporally constrained role for salience during saccadic choices (A) The compelled saccade task, an urgent color discrimination task. Subjects begin by fixating a central spot whose color, red or green, indicates the color of the search target (Fix). Two small gray placeholders appear next (Tar-On) separated by 180^o^. The disappearance of the fixation spot instructs the subject to respond (Go). Following a variable time gap (Gap, 25–250 ms), target and distracter identities are revealed (Cue). A trial is correct if the subject makes an eye movement (Saccade) to the location that matches the color of the initial fixation spot (green in this example) within 450 ms of the go signal (RT < 450 ms). Performance is dictated by the amount of time available for processing the visual cue in each trial (PT). (B) Example tachometric curve in the compelled saccade task (data from ref. 19, monkey T). Each point corresponds to the percentage of correct choices from all the trials within a processing time bin (bin size = 30 ms). Shading around traces indicates 95% confidence intervals from binomial statistics; dotted line denotes chance performance (50% correct); vertical line marks the approximate onset of rise in performance (129 ms). (C) The compelled antisaccade task. Subjects begin by fixating on a central spot (Fix), and its disappearance (Go) instructs the subject to make an eye movement. Following a variable time gap (Gap, 0–350 ms), the cue is revealed (Cue) to the left or the right (±10^o^). A trial is correct if the subject makes an eye movement away from the cue, to the diametrically opposite location (Saccade), within 450 ms of the go signal (RT < 450 ms). (D) Example tachometric curve in the compelled antisaccade task (data from ref. 12, results combined across 6 human participants). Same format as in B, with onset of rise at 114 ms.

The compelled saccade task (Figure 1A) is an urgent color-match task in which the instruction to respond (Go) is given prior to the cueing of task-relevant target information^14^^,^^15^^,^^17^^,^^18^^,^^19^ (STAR Methods). Subjects must respond immediately to this instruction in order to complete the trial within a maximum allowed reaction time (RT < 450 ms). After the go signal, following a variable time gap (Gap, 25–250 ms), target and distracter identities are revealed (Cue) and can guide the ongoing saccadic choice. Thus, time pressure compels subjects to plan—and sometimes execute—a movement before the relevant sensory information becomes available.

The parameter “gap” controls the difficulty of the compelled saccade task, but the likelihood of success in each trial is most strongly dictated by the amount of time the subject has to *process* the cue information prior to saccade onset. This processing time (**PT**) interval (Figure 1A) is obtained by subtracting the gap from the measured RT: PT = RT – gap, where all quantities are trial-specific. Then, as in other urgent tasks,^13^^,^^15^^,^^19^^,^^20^ perceptual capacity can be characterized via the tachometric curve, the function that tracks the percentage of correct responses as a function of PT (example in Figure 1B). Short PTs (⪅ 125 ms in this case) correspond to trials in which the subject was unable to resolve the color cue information (50% correct), whereas long PTs (⪆ 200 ms) correspond to trials in which the color cue was interpreted as per task rules and used to correctly locate the endogenously defined target (∼100% correct). The steepness of the transition from guesses to informed choices is an indicator of perceptual processing speed. Behavioral results in this task can be understood and quantitatively replicated by means of a race-to-threshold model that conforms to the motor selection dynamics measured in the frontal eye field during task performance.^14^^,^^17^^,^^18^^,^^21^

Performance in the compelled saccade task is driven by endogenous or top-down processes because the target is defined relative to the remembered fixation color and because the target and distracter are equally salient (or very nearly so; see STAR Methods). In contrast, in the compelled antisaccade task, performance depends on both exogenous (salience-driven) and endogenous (rule-driven) processes.^12^^,^^16^ In this version of the antisaccade task (Figure 1C), which is urgent, subjects are instructed to look away from a lone cue stimulus within 450 ms of the go signal. The tachometric curve (example in Figure 1D) again describes the evolution of a choice process that may result in a guess (at short PTs) or an informed decision (at long PTs), but now, within a narrow window of intermediate PTs, performance drops to nearly 0% correct. This corresponds to saccade plans that are inevitably captured by the cue.^22^^,^^23^^,^^24^ Such capture is fast, reliable, and sensitive to cue luminance^12^^,^^16^—properties characteristic of exogenous processes.^2^^,^^25^

The compelled saccade and compelled antisaccade tasks may seem very different. The former probes a decidedly visual capacity, color discrimination, whereas the latter is thought to summon cognitive resources specifically related to executive control and prefrontal activity.^26^^,^^27^^,^^28^^,^^29^^,^^30^^,^^31^^,^^32^ Indeed, impaired antisaccade performance is generally interpreted as a failure of inhibitory control.^33^^,^^34^^,^^35^^,^^36^ Against this conceptual framework, it is the similarities between the color matching and antisaccade tasks which stand out. In both, the target location is ultimately determined by an endogenous process that requires ∼200 ms of PT to approach asymptotic performance. For the former, the endogenously driven rise in accuracy (starting from chance) begins within ∼15 ms of the endogenously driven rise of the latter (starting from the lowest point of the tachometric curve), both near the 125 ms mark (Figures 1B and 1D, dashed lines). The exact numbers will depend on experimental conditions (e.g., stimulus luminance), but the point is that the rise in accuracy occurs within comparable time frames. Furthermore, the singular, non-monotonic shape of the antisaccade tachometric curve, together with other behavioral metrics, was comprehensively reproduced by a modified version of the race-to-threshold model for the compelled saccade task that was augmented with just one additional element, an exogenous response that transiently boosts the saccade plan toward the cue and suppresses the competing “anti” plan.^12^

All this raises the possibility that antisaccade performance fundamentally relies on the same visuomotor mechanisms that support saccadic choices in general—except with a particularly strong exogenous contribution.

### Model predictions for a new task in which salience varies but is irrelevant

**Figure 2:**
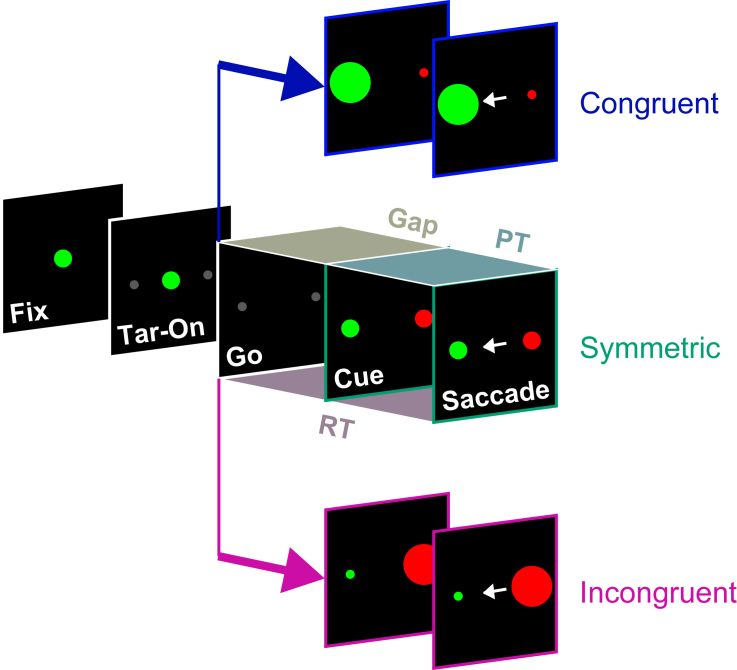
The color matching with varying salience (CMVS) task The basic structure and rule set of the CMVS task are the same as for the compelled saccade task (Figure 1A). The subject fixates on a central spot (Fix) whose color, red or green, indicates the color of the search target. After the two placeholders appear (Tar-On), the disappearance of the fixation spot instructs the subject to respond (Go). Following a time gap (Gap, 25–250 ms), target and distracter identities are revealed (Cue). A trial is correct if the subject makes an eye movement (Saccade) to the location that matches the color of the initial fixation spot (green in these examples) within 450 ms of the go signal (RT < 450 ms). The same color-match rule applies to the three trial types, which vary by cue salience. Top row: a congruent trial; the target is the salient (larger) item. Middle row: a symmetric trial; the target and distracter are equally salient. Bottom row: an incongruent trial; the distracter is the salient (larger) item. Trial types are always interleaved. The probability of success depends critically on the amount of cue processing time (PT) in each trial.

To investigate the above hypothesis, we sought to characterize the effect of uninformative exogenous signals on developing, endogenously defined saccadic goals. As such, subjects in this study were not instructed to look away from stimuli or withhold any responses; rather, all trials adhered to the same color-match rule set. In the new color matching with varying salience (**CMVS**) task, physical salience varies across stimuli but provides no information about target identity (Figure 2). Three trial types are interleaved: symmetric trials with same-size cues (Figure 2, middle row), which are identical to those in the original compelled saccade task (Figure 1A); congruent trials, in which the salient, larger item is the target (Figure 2, top row); and incongruent trials, in which the salient, larger item is the distracter (Figure 2, bottom row).

If our hypothesis is correct, behavior should be dictated by the same dynamics as in the compelled antisaccade task, except that the exogenous contribution, which simply tracks salience, may now reinforce the target (in congruent trials), the distracter (in incongruent), or neither (in symmetric). Thus, we (slightly) modified the existing model for the urgent antisaccade task so that it would simulate the three CMVS trial types in accordance with this hypothesis (STAR Methods).

**Figure 3:**
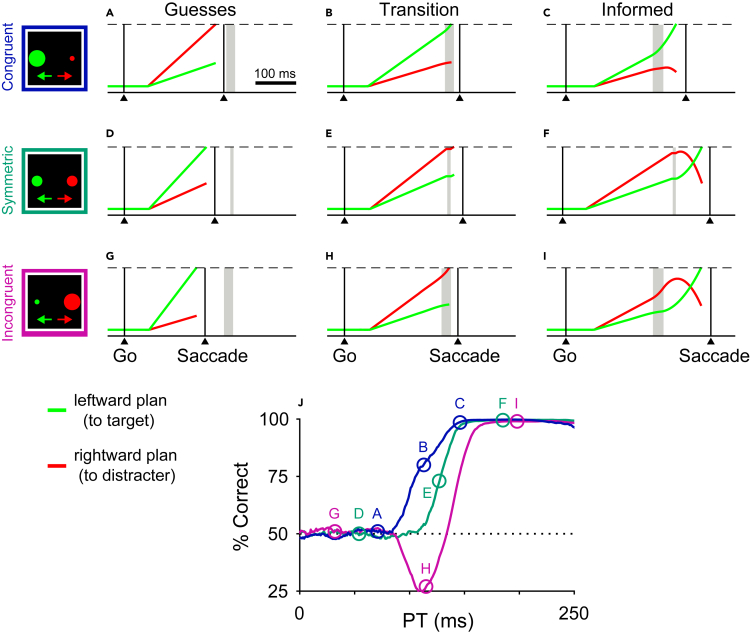
The race-to-threshold model and its prediction of performance in the CMVS task (A–I) Model single trials. Each panel shows competing motor plans from two neuronal populations attempting to trigger eye movements toward left (green) or right (red) stimulus locations. Each trace represents neuronal activity as a function of time. The first plan to reach the fixed threshold (dashed lines) triggers a saccade in its preferred direction. Icons indicate target (green) versus distracter (red) salience across trial types. In all examples, the target is on the left, so correct and incorrect outcomes correspond to races that are won by the green and red traces, respectively. Gray shades represent the cue detection period (ERI), during which neuronal responses are modulated exogenously. In congruent trials (A–C), the ERI modulation favors the target, which is larger/more salient. In symmetric trials (D–F), both neuronal responses pause briefly during the ERI. In incongruent trials (G–I), the ERI modulation favors the distracter, which is larger/more salient. After the ERI, the cue content is fully resolved, so the plan toward the target (correct) accelerates and that toward the distracter (incorrect) decelerates. Trials that reach threshold prior to the ERI (A, D, G) are guesses and have short PTs. Trials that reach threshold after cue resolution (C, F, I) are informed and have long PTs. Trials that reach threshold during or just after the ERI (B, E, H) are partially informed and have intermediate PTs. The outcome of each trial ultimately depends on the initial build-up rates of the motor plans and the time at which the cue information arrives (in all examples, the gap is 150 ms). (J) Simulated tachometric curves for congruent (blue), symmetric (teal), and incongruent (magenta) trial types. Circles represent PTs for all the single-trial examples. The dotted line marks chance level (50% correct).

The model (Figure 3) universally considers two motor plans, i.e., population activities promoting specific saccades, one to a right location (Figures 3A–3I, red traces) and another to a diametrically opposite left location (green traces). Following the go signal (Figures 3A–3I, left vertical lines) and an afferent delay, the motor plans rise at variable rates based on intrinsic bias, attentional allocation, or other internal factors, and the first plan to reach a fixed threshold (dashed lines) triggers the ensuing saccade to the left or to the right (right vertical lines). Because the cue is revealed after the go signal, the cue information may or may not arrive in time to guide the choice process. When the cue information arrives late, after the threshold has been crossed, the triggered saccade is uninformed, i.e., a guess (Figures 3A, 3D, and 3G). However, when the information arrives early, it modulates the ongoing plans in two temporally and qualitatively distinct ways. First, the cue is detected but cannot yet be used in an endogenous manner. During this period, called the exogenous response interval (**ERI**; Figures 3A–3I, vertical gray shades), motor plans to spatial locations that are congruent with salient events are transiently accelerated (Figures 3B and 3C, green traces; Figures 3H and 3I, red traces) while the opposing motor plans are halted or suppressed (Figures 3B and 3C, red traces; Figures 3H and 3I, green traces). Then, following the ERI, once the cue content has been interpreted according to the color-match rule set, it can endogenously guide the choice process. Thus, once the target has been identified, the plan toward it is accelerated (Figures 3C, 3F, and 3I, green traces) while the plan toward the distracter is decelerated (red traces).

Across the three CMVS trial types, the only difference in these dynamics is that in the symmetric case (Figures 3D–3F), the ERI is shorter and the symmetric exogenous response does not favor either plan, consistent with the two stimuli having equal salience. Aside from this, the model was always run with identical parameter values for the three task conditions.

Note that the model does not pretend to explain how or where the perceptual decision signals are generated; rather, it simply assumes that at some point such signals modulate the oculomotor activity that reports the choice—as they must. Critically, however, the sign, magnitude, and timing of these perceptually driven modulations are intimately related to the behavioral metrics observed in urgent tasks^12^^,^^14^^,^^16^^,^^17^ and, in particular, to the shape of the resulting tachometric curve(s). In the current case, this leads to clear differential predictions (Figure 3J).

At short PTs (< 80 ms), performance is the same for all trial types: the motor plan with the higher initial buildup rate reaches the threshold first and triggers an uninformed saccade—that is, a guess (Figures 3A, 3D, and 3G). At intermediate PTs (approximately 80–130 ms), motor plans that are near the threshold and congruent with a salient stimulus may be accelerated past the point of commitment, resulting in exogenously triggered saccades (Figures 3B and 3H). For congruent trials (salient target), such captured saccades are correct choices (Figure 3B) and promote an early rise of the tachometric curve (Figure 3J, blue trace), whereas for incongruent trials (salient distracter), they correspond to errors (Figure 3H) that initially drive performance below chance and delay the rise of the tachometric curve (Figure 3J, magenta trace). Finally, at long PTs (> 130 ms) the endogenous modulation reverses competitions that would otherwise lead to errors (Figures 3F and 3I), thus populating the asymptotic portion of the tachometric curves.

The predicted tachometric curves (Figure 3J) were qualitatively the same within a vast parameter range (STAR Methods). In the symmetric case (teal trace) there is no capture. When the distracter is more salient than the target (incongruent case; magenta trace), performance is similar to that in the antisaccade task, with a dip below chance and a rise in accuracy that lags that in the symmetric trials. And when the target is more salient (congruent case; blue trace), accuracy rises sooner than in the symmetric version of the task. Finally, the congruent and incongruent curves should initially depart from chance in different directions but at the same time point, because the underlying exogenous mechanism is the same.^16^

### Saccadic choices are always biased toward higher salience

**Figure 4:**
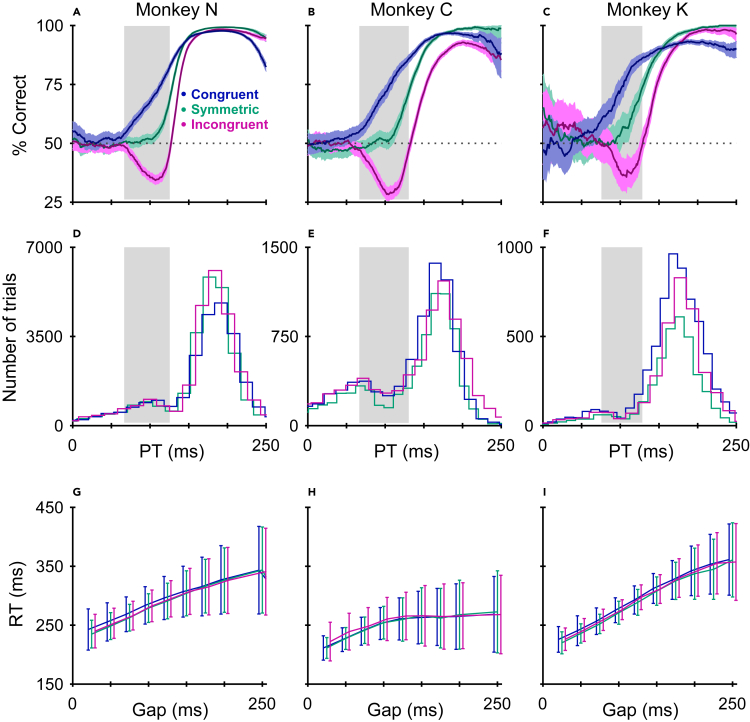
Behavioral data from three monkeys in the CMVS task (A–C) Tachometric curves showing the percentage of correct responses as a function of PT for congruent (blue), symmetric (teal), and incongruent (magenta) trial types. Each point corresponds to the percentage of correct choices from all the trials within a 50 ms PT bin. Shading around traces indicates 95% confidence intervals from binomial statistics, dotted lines denote chance performance (50% correct), and gray shades mark the 75–125 ms PT interval during which saccades are typically captured exogenously. (D–F) Histograms showing number of trials collected in non-overlapping PT bins. (G–I) Mean RT ± 1 SD as a function of gap duration. Data points are based on trials pooled across experimental sessions. (A, D, G) Data from monkey N (25,303 congruent, 25,889 symmetric, 26,206 incongruent trials). (B, E, H) Data from monkey C (7901 congruent, 7903 symmetric, 8115 incongruent trials). (C, F, I) Data from monkey K (6313 congruent, 3476 symmetric, 3473 incongruent trials).

Three monkeys performed the CMVS task with the three trial types randomly interleaved (STAR Methods), and the results were highly consistent with the model predictions (Figure 4).

In trials with PTs shorter than 75 ms, the subjects performed at chance level, as expected given such limited amount of cue viewing time. For PTs of ∼75–125 ms (Figures 4A–4C, gray shades), choice accuracy demonstrated an early improvement in the congruent condition, as subjects directed a sizable proportion of their saccades to the salient target (Figures 4A–4C, blue traces). In contrast, in this same PT range, choice accuracy dipped below chance in the incongruent condition, as subjects often made erroneous saccades to the salient distracter (Figures 4A–4C, magenta traces). Note the large separation between error bands (indicating 95% confidence intervals) in this range. Thereafter, for PTs beyond ∼125 ms, the success rate reliably approached an asymptote in all conditions, suggesting that by that point endogenous mechanisms were already guiding the appropriate motor response according to the color-match rule. In the symmetric CMVS trials, the tachometric curve displayed the same characteristic time course observed in earlier studies^14^^,^^15^^,^^17^^,^^19^ (Figures 4A–4C, teal traces; compare to Figure 1B); it fell squarely between the two asymmetric-salience cases, as predicted.

The early, exogenous impact of the cue onset is most evident by comparing the three psychometric functions at around the 110 ms mark. By this point, performance in the symmetric trials, in which salience was even, is just starting to rise above chance (Figures 4A–4C, teal traces), presumably driven by endogenous mechanisms exclusively. In contrast, after the very same amount of processing time, performance in the incongruent trials is near its minimum, well below 50% correct, whereas performance in the congruent trials is around 75%—both deviations driven by essentially the same exogenous signal, according to the assumption underlying the model predictions.

All these results were qualitatively the same across subjects, as well as for choice stimuli presented at different orientations and eccentricities (STAR Methods). They were also highly robust with respect to the number of trials performed by each monkey (Figures 4D–4F).

As noted previously,^14^^,^^15^^,^^17^^,^^19^^,^^37^ the urgent-choice design decouples perceptual and motor performance such that the tachometric curve is largely impervious to variance in motor planning. As a consequence, it is possible to observe large variations in the tachometric curve without any changes in the chronometric (RT) curve, and vice versa. Such independence was evident in the current dataset as well. For each monkey, the mean and SD of the RT as functions of gap were essentially indistinguishable across the three task conditions (Figures 4G–4I), in spite of obvious differences in choice accuracy (Figures 4A–4C). Conversely, in the three monkeys, the three tachometric curves were similarly related to each other, even though one animal tended to respond much more quickly (∼40 ms) than the other two (mean RTs were 289 ± 53 ms, 251 ± 46, and 294 ± 57 for monkeys N, C and K, respectively; mean ± SD, with SEM values of approximately 1 ms). This confirms that the tachometric curve can be reliably interpreted as a stand-alone assessment of perceptual performance that is minimally susceptible to speed-accuracy tradeoffs.^14^^,^^15^^,^^17^^,^^19^^,^^37^

**Figure 5:**
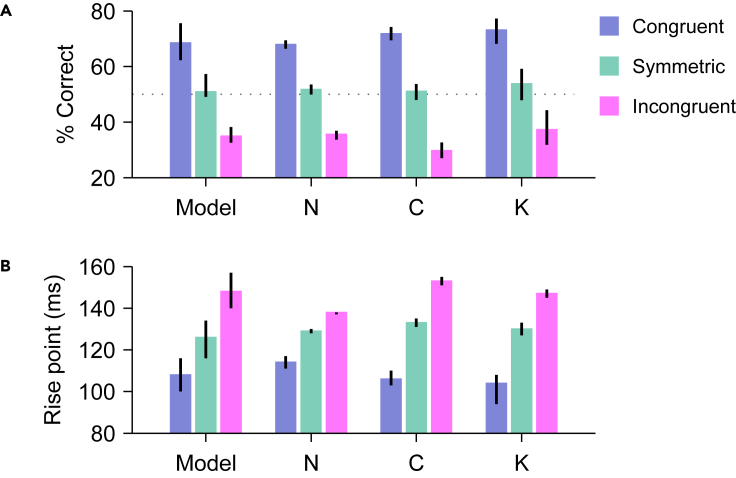
Comparison between model and experiment (A) Mean fraction correct in the capture range. Results are shown for congruent (blue), symmetric (teal), and incongruent trials (magenta) from model simulations (Model) and from the three monkeys (N, C, and K). In each case, percent correct was computed from all the trials with PTs in the 75–125 ms range (gray shades in Figures 4A–4C). For the monkey data, error bars indicate 95% confidence intervals from binomial statistics. For the model, error bars indicate 95% confidence intervals across simulations with randomized parameters (SD = 5% for each parameter value; see STAR Methods). (B) Rise point in performance. The rise point is the PT at which the tachometric curve reaches 75% correct. Format is the same as in A. For the monkey data, error bars indicate 95% confidence intervals from bootstrap. For the model, error bars indicate 95% confidence intervals across simulations with randomized parameters (SD = 5% for each parameter value; see STAR Methods).

Finally, to more directly compare the experimental results to the model predictions, we calculated two quantities. The first one is the mean percentage of correct choices in the range where exogenous capture is highly likely (75 ≤ PT ≤ 125 ms), and measures the strength and direction of the exogenous influence. The second is the rise point of the tachometric curve, i.e., the PT at which performance first exceeds 75% correct, and measures when the saccadic choices become reliably informed. As judged by both quantities, the impact of salience was highly consistent across the three monkeys and conformed closely to the model predictions (Figure 5). As expected, captured saccades manifested as large deviations above and below 50% correct for salient targets and salient distracters, respectively (Figure 5A), and the rise toward asymptotic performance was advanced or delayed accordingly (Figure 5B). In simulations with random variations in parameter values (STAR Methods), the model produced sets of results that were qualitatively similar, and their range overlapped highly with the experimental data (Figure 5, note error bars for Model; see caption).

## Discussion

We developed an urgent color-match task with varying salience to investigate the automaticity of exogenously driven responses and their interaction with endogenous perceptual signals during saccadic choices. By dissociating the target/distracter designation (defined by color) from object salience (manipulated through size), we were able to verify model predictions based on a specific functional hypothesis: that the initial detection of a visual cue by oculomotor circuits always favors the selection of salient items regardless of their behavioral relevance. Thus, exogenous mechanisms boosted performance when the target was salient but hindered it when the distracter was salient—a rapid, involuntary effect that was quickly superseded by endogenous mechanisms.

Because of the high temporal resolution afforded by the urgent task design,^13^^,^^15^ we were able to show that this exogenous-endogenous alignment effect is not only strong and reliable, but also remarkably brief: the exogenous response interval in the model lasted ∼25 ms (Figures 3A–3I), and empirically, the exogenous capture occurred over a window of ∼50 ms (Figures 4A–4C). These two properties reconcile somewhat contradictory sets of observations. On one hand, the presence of a salient distracter does not necessarily lead to overt capture; it can manifest as a subtle difference in RT during standard, non-urgent laboratory tasks,^9^^,^^24^^,^^38^ or may become altogether undetectable during even more protracted, naturalistic tasks.^6^^,^^7^^,^^8^ This is consistent with the short-lived nature of the exogenous influence. On the other hand, the highly resilient effect of exogenous signals on motor responses is apparent in a variety of behavioral and neurophysiological measurements taken over relatively short time scales.^13^ For instance, analyses of microsaccades show that exogenous events have direct modulatory access to ongoing oculomotor activity during fixation.^39^^,^^40^^,^^41^ Specifically, a stimulus that appears within approximately ±30 ms of a microsaccade has a stereotypical impact on its amplitude and velocity, and the effect can be tightly related to the activity of visual and motor neurons in the superior colliculus.^42^ Such interactions between exogenous signals and motor plans are most likely facilitated by the overall functional layout of oculomotor areas, which contain local, visually sensitive neurons that communicate salience information at very low latencies^43^^,^^44^ and can quickly influence eye movements.^42^^,^^45^^,^^46^ Indeed, single-neuron recordings in the superior colliculus and frontal eye field suggest that, during antisaccade errors, the visual response to the cue onset propels ongoing oculomotor activity beyond the threshold for triggering a movement,^47^^,^^48^ consistent with our model (Figure 3). Finally, the resilience of exogenous signals and their likely association with attentional or oculomotor capture is also revealed in experiments with reaching tasks. When a novel visual stimulus appears, a rapid stimulus-locked response is detectable in the muscles of the neck and upper body via electromyography (EMG) recording.^49^^,^^50^ This activity has all the properties expected of an exogenous response: short latency, short duration, dependence on salience, invariance to task rules, and contribution to “express” movements. And notably, during anti-reaches, this activity tracks the stimulus properties (onset, location, and salience) and is predictive of correct versus incorrect outcomes.^49^

The close correspondence between the CMVS incongruent condition in the current experiment and the urgent version of the antisaccade task^12^^,^^16^ suggests that common neural mechanisms drive behaviors that may seem to tap very different cognitive resources. The antisaccade task is traditionally considered a sensitive assay of top-down, inhibitory control processes that mediate, via prefrontal circuits, the voluntary suppression of unwanted actions.^26^^,^^27^^,^^28^^,^^29^^,^^30^^,^^31^^,^^32^ However, under urgent conditions the majority of antisaccade errors are quite stereotypical: they depend strongly on stimulus luminance and correspond to saccades that are captured very rapidly, within 80–130 ms of cue onset.^12^ Moreover, the underlying exogenous process responsible for this involuntary capture seems to be fundamentally identical when a prosaccade instead of an antisaccade is instructed.^16^ The results obtained here provide critical support for such low-level interpretation because they demonstrate the very same dependence on salience but without explicitly requesting a movement away from a stimulus, or requiring the suppression of any particular action.

This is not to deny the participation of inhibitory or other specific mechanisms (top-down, prefrontal) in antisaccade performance, but rather to note that, whatever their involvement, it must be much wider than is generally assumed. The compelled antisaccade task can be considered an extreme version of the CMVS incongruent condition wherein the size of the target approaches zero. The qualitative similarity in performance profiles, and the success of the same model in reproducing them quantitatively, suggests that there is no fundamental difference between the problem of looking away from a conspicuous stimulus (antisaccade trials) and the problem of looking at an inconspicuous one (CMVS incongruent trials).

The same type of conflict between salience and endogenous goals has also been documented in the context of reaching movements using a “go-before-you-know” task design that in many ways resembles our own urgent paradigms.^51^ Using this technique, Wood and colleagues showed^52^ that when the potential reach targets vary in luminance, the initial uninformed trajectory is biased toward the high salience item even when that option has a lower probability of being the correct choice. In that case, the spatial trajectory of the hand over time (within a trial) evolves in a manner that is directly analogous to the evolution of the saccadic choice over processing time as described by the tachometric curve in the incongruent condition (Figures 4A–4C, magenta curves).

The generality of the underlying computational task, i.e., overcoming the automatic draw of salient stimuli, becomes even more evident when considering that the concept of salience likely extends beyond physical qualities such as size, motion, or luminance. Non-salient stimuli have been shown to produce involuntary capture when previously associated with reward,^53^ when predicting a shock,^54^ or when they conflict with the goal of an ongoing urgent choice^20^—in the latter case, producing tachometric curves with a pronounced dip like those in the incongruent and antisaccade tasks. Thus, the phenomenology of the antisaccade task is likely a somewhat extreme product of normal visuomotor dynamics that operate under many circumstances. From this perspective, it is hardly surprising that antisaccade performance is linked to activation in numerous and diverse brain areas,^28^^,^^55^^,^^56^^,^^57^ or that it correlates with performance in many other behavioral tests.^30^^,^^58^^,^^59^

The results suggest that the problem of how to combine exogenous information (i.e., sensory properties) and endogenous signals (i.e., internal goals) to guide motor choices is a fundamental one and that it likely summons similar mechanisms during behaviors that vary widely in their complexity and specifics.

### Limitations of the study

In our experiment, salience differences were generated by introducing asymmetries in stimulus size, but as mentioned previously, the interactions between intrinsic features and surrounding context that define perceived salience are complex. For instance, in addition to the spatial and feature contributions capable of creating pop-out effects (e.g., a red apple atop a pile of green apples), variations over time can also have a strong impact on salience that may be hard to predict.^60^^,^^61^^,^^62^^,^^63^ Thus, although our current and prior results suggest that qualitatively similar exogenous capture should be observed whenever salience favors some objects more than others, predicting the strength of the ensuing effects may be difficult. In other words, our data reveal that exogenous signals are very brief and rather impervious to voluntary control, but do not clarify the question of what determines their magnitude.^10^

## STAR★Methods

### Key resources table

**Table:** 

REAGENT or RESOURCE	SOURCE	IDENTIFIER
**Deposited data**		

Behavioral data	This work; Zenodo	https://doi.org/10.5281/zenodo.7342804

**Software and algorithms**		

MATLAB and Statistics Toolbox	Mathworks	https://www.mathworks.com/products/matlab.html
Gramalkn	Ryklin Software	https://ryklinsoftware.com/
Race-to-threshold model	This work; Zenodo	https://doi.org/10.5281/zenodo.7343309

### Resource availability

#### Lead contact

Further information and requests for resources should be directed to and will be fulfilled by the lead contact, Emilio Salinas (esalinas@wakehealth.edu).

#### Materials availability

This study did not generate new unique reagents.

### Experimental model and subject details

#### Subjects

Data were collected from three purpose-bred adult male rhesus monkeys (*Macaca mulatta*, from World-Wide Primates, Miami, FL). They were 7–11 years old and weighed 10–19 kg at the time of data collection. All subjects met veterinary standards for health quality upon arrival at Wake Forest School of Medicine (WFSM) and were monitored daily thereafter by veterinary and lab staff. Monkeys were pair-housed in communal rooms and provided sensory enrichment in addition to interactions with other monkeys. Prior to the current study, two animals (monkeys N and C) had served as experimental subjects in related studies with urgent tasks and one animal (monkey K) was naive. In preparation for behavioral training, an MRI compatible head post was secured to the skull of each animal using standard sterile surgical techniques under general anesthesia. The opioid analgesic Buprenorphine hydrochloride was used to manage post-surgical pain and the NSAID Ketoprofen was used to manage post-surgical inflammation. Following a recovery period of ∼2 weeks, animals were familiarized with head fixation prior to instrumental training.

All experimental procedures were compliant with the NIH Guide for the Care and Use of Laboratory Animals, USDA regulations, and the guidelines put in place by the Institutional Animal Care and Use Committee (IACUC) of Wake Forest School of Medicine, an institution accredited by the Association for Assessment and Accreditation of Laboratory Animal Care International (AAALAC International), under protocols A16–090 and A19-068.

### Method details

#### Training procedure

Using standard operant conditioning, head restrained subjects were trained to perform the color matching with varying salience (CMVS) task (Figure 2) via the delivery of a liquid reward and a secondary reinforcement sound after correct completion of each task trial. On working days, trained animals were water restricted in their home cages and the performance of visual tracking tasks served as their primary source of hydration. Behavioral recording sessions were conducted until animals presented signs of satiation (decreased motivation in latter stage of session). In the event that the minimum daily water requirements specified by WFSM policy were not met, supplementary liquid was provided to the animal in their home cage post-session. Food was provided *ad libitum* to the animals in their home cages by the WFSM Animal Resources Program (unless otherwise specified by veterinary staff). Supplemental food and enrichment were provided daily by laboratory staff.

#### Behavioral recording techniques

In a quiet, dimly lit room, colored visual stimuli were presented on a VIEWPixx LED monitor (VPixx Technologies Inc, Saint Bruno, QC, Canada; 1920 × 1200 screen resolution, 120 Hz refresh rate, 12 bit color) at a viewing distance of 57 cm from the eyes of head-restrained subjects. An infrared camera-based eye tracking system was used to sample eye movements at 1000 Hz (EyeLink 1000, SR Research, Ottawa, Canada). Stimulus presentation and data acquisition were controlled via the custom-designed Gramalkn software (Ryklin Software Inc., New York, NY).

#### Behavioral task

In the previously developed compelled saccade task, subjects were required to look at the stimulus that matched the color of the fixation spot under time pressure.^14^^,^^17^^,^^18^^,^^19^ In the new CMVS task, all three trial types followed the same structure and color match-to-sample rule set, but with stimuli of varying sizes.

Subjects began by fixating a central spot that appeared on a black background and whose color — red or green — signaled the color of the search target (Figure 2, Fix). While maintaining fixation, two dark gray placeholders (0.5^o^ diameter) spaced 180° apart, equidistant from fixation, were presented to indicate the locations of the future target and distracter (Figure 2, Tar-On). After a delay of 250–550 ms, the disappearance of the fixation spot (Figure 2, Go) instructed the subjects to make an eye movement, and it had to be initiated within 450 ms for a chance at a liquid reward; that is, the maximum reaction time (RT) allowed was 450 ms (Figure 2, RT). Target and distracter identities were revealed (Figure 2, Cue) following a variable time interval of 25–250 ms (Figure 2, Gap). A trial was correct when the subject made an eye movement to the stimulus that matched the color of the initial fixation spot within the 450 ms of allotted time. Correct trials were rewarded with a drop of liquid and a white-noise burst. Typically, blocks of 100 trials with a red target and green distracter were interleaved with comparably sized blocks containing a green target and red distracter.

Three trial types — in which the salience of the stimuli varied independently of the target-distracter designation — were randomly interleaved. In congruent trials (Figure 2, top), the target was larger (diameter = 2.5^o^ of visual angle) and thus more salient than the distracter (0.5^o^ diameter). In incongruent trials (Figure 2, bottom), the target was less salient (0.5^o^ diameter) than the distracter (2.5^o^ diameter). And in symmetric trials (Figure 2, middle), the target and distracter were equally salient (1.0^o^ diameter). Across experimental sessions, choice stimuli were presented at different orientations (approximately 33% near horizontal, 15% near vertical, and 52% near diagonal) and eccentricities (median of 11^o^, range 5–20^o^, 90% range 7–18^o^).

Data from monkeys N, C, and K were collected in 89, 39, and 14 experimental sessions (i.e., separate days), respectively. These sessions spanned 13, 5, and 2 months, and had a median of 863 ([412, 1441] 95% confidence interval), 575 ([78, 1203] 95% confidence interval), and 857 ([532, 1661] 95% confidence interval) trials recorded per session, bringing the total numbers of trials to 77,398, 23,919, and 13,262.

### Quantification and statistical analysis

#### Analysis procedure

Data were analyzed using customized scripts in MATLAB R2020a (The MathWorks, Natick, MA). In each trial, the RT was measured as the interval between the go signal and saccade onset, defined as the time at which eye velocity surpassed 50^o^/s.

Performance in our urgent tasks is dictated by the processing time (PT), which is the maximum amount of time during which subjects can view and analyze the cue before initiating their saccadic response. This corresponds to the interval between cue onset and saccade onset (Figure 2, PT), and was determined by subtracting the duration of the main control parameter “gap” from the RT measured on each trial. That is, via

PT=RT−gap

where all quantities are specific to each trial. To evaluate the degree to which the color cue informs the subject’s decisions, the percentage of correct choices was plotted as a function of PT to form a psychometric function that we refer to as the tachometric curve.^12^^,^^14^^,^^15^^,^^16^^,^^17^^,^^18^^,^^19^ Tachometric curves for each trial type were computed by aggregating trials across experimental sessions and sorting them into 50 ms PT bins that shifted every 1 ms. The binofit function from the MATLAB Statistics Toolbox was used to determine 95% confidence intervals for these curves.

Since targets within the CMVS task could be either green or red with an oppositely colored distracter, performance in each trial type was first evaluated separately for each color. Analogous behavioral patterns were found when the target color was red versus when the target color was green, so trials were combined for analysis. Because behavioral patterns were also qualitatively the same regardless of stimulus eccentricity and orientation, the data were pooled accordingly.

#### The accelerated race-to-threshold model

The model for the CMVS task was derived from the model developed for the original compelled saccade task^14^^,^^17^^,^^18^ and its more recent extension to the compelled antisaccade task.^12^^,^^16^ The version of the model implemented here is fundamentally the same as the latter, except that during the exogenous response interval (ERI; Figures 3A–3I, gray shaded areas), the modulations applied to the two motor plans are assigned depending on the salience of the corresponding choice stimuli, so they can favor either the target or the distracter.

The motor competition model has two variables, $x_{L}$ and $x_{R}$, that represent the population activity of neurons triggering movements to the left and to the right, respectively. These variables increase over time, racing to be the first to reach a fixed threshold. If $x_{L}$ wins, the outcome of the trial is an eye movement to the left; if $x_{R}$ wins, the outcome is an eye movement to the right. The competition begins after the go signal, which occurs at t=0, following an afferent delay drawn from a Gaussian distribution. Thereafter the motor plans evolve according to their build-up rates, *b*_*L*_ and *b*_*R*_, such that

$$x_{L}\left(t+\Delta t\right)=x_{L}\left(t\right)+b_{L}\Delta t$$

$$x_{R}\left(t+\Delta t\right)=x_{R}\left(t\right)+b_{R}\Delta t$$

where Δt is the integration time step (= 1 ms). Initially, the activities ramp up at constant rates $b_{L}^{0}$ and $b_{R}^{0}$ drawn from a two-dimensional Gaussian distribution with mean $\mu_{b}$, SD $\sigma_{b}$, and correlation coefficient $\rho_{b}$. This corresponds to the internally driven preparation for making a response. If during this stage one of the motor plans reaches the threshold, a saccade is produced to the winning direction constituting a random guess. The above equations always dictate the evolution of the motor plans. Later events influence the selection process by altering the build-up rates *b*_*L*_ and *b*_*R*_.

Urgent paradigms are structured such that the cue is revealed gap milliseconds after the go signal. Cue information subsequently reaches the model circuit at $t=gap+T_{a}$ where $T_{a}$ is the afferent delay of cue detection. This marks the beginning of the ERI, whose duration varies normally across trials; it is drawn from a Gaussian distribution with mean $\mu_{ERI}$ and SD $\sigma_{ERI}$. It is during the ERI that the model dynamics for the three trial types diverge. In symmetric trials, as in the original model,^14^^,^^17^ both motor plans halt (or slow down) briefly during the full ERI (Figures 3E and 3F), consistent with the simultaneous presentation of a target and distracter that are equally salient. This halting/slowdown is implemented by setting the build-up rates to $b_{L}=g_{ERI}b_{L}^{0}$ and $b_{R}=g_{ERI}b_{R}^{0}$, during this period, where $g_{ERI}$ is a gain factor between 0 and 1. In congruent and incongruent trials, the two plans also halt (or slow down) during the initial $\Delta_{ERI}$ ms of the ERI, with their build-up rates again attenuated by a factor $g_{ERI}$, but the plans then evolve differently during the remainder of the ERI. During that time, the dynamics depend on the location of the salient versus the non-salient item, so the corresponding activity variables are re-labelled $x_{S}$ and $x_{N}$, respectively. With this notation in mind, the motor plan toward the non-salient item ($x_{N}$) keeps halting or progressing more slowly (so $b_{N}=g_{ERI}b_{N}^{0}$), whereas the motor plan toward the salient item ($x_{S}$) accelerates, which means that its build-up rate increases linearly over time, such that

$$b_{S}\left(t+\Delta t\right)=b_{S}\left(t\right)+a_{EX}\Delta t$$

where $a_{EX}$ is the exogenous acceleration of the salient item.

Again, note the intentional exclusion of a target versus distracter argument. In the congruent trial type, $x_{S}$ would correspond with the location of the target while $x_{N}$ would correspond with the location of the distracter (Figures 3B and 3C). However, in the incongruent case, $x_{S}$ would now correspond with the location of the distracter and $x_{N}$ with that of the target (Figures 3H and 3I). If the exogenous acceleration drives $x_{S}$ to reach the threshold, a saccade is triggered toward the salient item regardless of its target/distracter identity. Because at this stage of the model the color information is yet to be integrated with the endogenous rule-set, any cue-driven change in activity during this period (ERI) represents an exogenous effect.

Following the ERI, the model responses are modulated according to the locations of the color-defined target and distracter. In other words, once the color cue has been resolved and interpreted, the choice evolves toward a successful outcome: the (correct) motor plan to the target location accelerates while the (erroneous) plan to the distracter decelerates. This means that, in the congruent trial type, $x_{S}$ accelerates and $x_{N}$ decelerates (Figure 3C),

$$b_{S}\left(t+\Delta t\right)=b_{S}\left(t\right)+a_{END}\Delta t$$

$$b_{N}\left(t+\Delta t\right)=b_{N}\left(t\right)+d_{END}\Delta t$$

whereas in the incongruent trial type, the association is reversed, so $x_{N}$ accelerates and $x_{S}$ decelerates (Figure 3I)

$$b_{N}\left(t+\Delta t\right)=b_{N}\left(t\right)+a_{END}\Delta t$$

$$b_{S}\left(t+\Delta t\right)=b_{S}\left(t\right)+d_{END}\Delta t$$

Here, $a_{END}$ is a positive term representing the endogenous acceleration of the target plan and $d_{END}$ is a negative term representing the endogenous deceleration of the distracter plan. Finally, in symmetric trials, if the target appeared on the left, as in the examples depicted throughout the main text, then $x_{L}$ accelerates and $x_{R}$ decelerates post-cue resolution (Figure 3F). The converse applies in the reverse situation, when the target is on the right and distracter on the left. In either case, the relevant equations are analogous to those above, but with corresponding *L* and *R* subscripts.

Whenever a plan reaches threshold, a saccade to the winning direction is considered to be triggered following a short, fixed efferent delay. Finally, the model includes the parameter λ, which represents the probability of a lapse, i.e., of making an error independently of the processing time. When a lapse occurs, the endogenous acceleration and deceleration are never applied, as if the cue colors had never been resolved; $a_{END}$ and $d_{END}$ are set to zero in this case, and the motor plans continue evolving with the build-up rates achieved at the end of the ERI until one of them exceeds the threshold.

For Figure 3, parameter values were adjusted, based on prior results,^12^^,^^16^ to produce a moderate amount of exogenous capture in the incongruent condition. For Figure 5, random variations around this same parameter set were generated as follows. In each run of the model, which comprised 40,000 simulated trials, the i’th model parameter was equal to

$$p_{i}=\left(1+0.05\varepsilon \right)$$

where $p_{i}$ is the base value used in Figure 3 and ε is a normally distributed random number different for each parameter and each run. This way, each parameter had an SD equal to 5% of its magnitude when sampled across runs, and parameters were randomized independently. The error bars shown in Figure 5 for the model are 95% confidence intervals obtained across 500 model runs. They are indicative of the range of tachometric curves that the model can generate based on such simultaneous parameter variations.

MATLAB code for running the model, visualizing the results, and comparing them to the experimental data are included as part of the shared data and code packages. The DOIs are listed in the key resources table.

## Data Availability

Trial-by-trial behavioral data have been deposited in the Zenodo repository and are publicly available. The DOI is Zenodo: https://doi.org/10.5281/zenodo.7342804, also listed in the key resources table. MATLAB scripts for reproducing experimental results are included as part of the shared data package. All original code (for the race-to-threshold model) has been deposited at Zenodo and is publicly available. The DOI is Zenodo: https://doi.org/10.5281/zenodo.7343309, also listed in the key resources table. Any additional information required to reanalyze the data reported in this paper is available from the lead contact upon request.
